# Hepatitis B Infection during Renal Replacement Therapy

**Published:** 2010-12-01

**Authors:** Sanjay Kumar Agarwal

**Affiliations:** 1 Department of Nephrology, AIIMS, New Delhi, India

Hepatitis B virus (HBV) infection in patients with end-stage renal disease (ESRD) on renal replacement therapy-hemodialysis and/or renal transplantation-usually has an unfavorable course with a tendency towards chronicity leading to increased morbidity and mortality [1]. Furthermore, the patients with the infection remain a source of nosocomial infection during hemodialysis. Though the incidence of HBV infection in patients with ESRD has significantly decreased due to preventive measures like vaccination and obedience to universal precaution and isolation rules during dialysis, this infection still continues to occur during renal replacement therapy in many units in the world including India. Patients with ESRD remain at increased risk of contracting HBV because of increased exposure to blood products, shared hemodialysis equipment, frequent breaching of the skin, immunodeficiency of Chronic Kidney Disease (CKD), and continuing high prevalence rates of HBV infection among hemodialysis patients. The frequency of HBV seropositivity in renal replacement therapy in India had been reported from 4% to 44% [2][3][4]. Higher frequencies are reported only from few units, while in most of the larger well-organized units, it is less than 5%. Patients with ESRD regularly get transplant unless they have significant histological fibrosis or clinically advanced liver disease. We had earlier reported that chronic liver disease, due to hepatitis, was the second most common cause of death in renal transplant patients in the second decade post-transplant [5].Universal precautions are the most accepted method for prevention of blood-borne infections in hemodialysis program, though it is easier said than done. Many units could not demonstrate decrease in the rate of blood-borne infection while claiming to follow universal precautions. Obviously, there is gap between knowing the principles of the precautions and implementing them in day to day practice. In various types of blood-borne infections, HBV infection has a different scenario than others like hepatitis C virus (HCV) and human immunodeficiency virus (HIV) infection. HBV prevention is possible by vaccination and abiding to universally-accepted approaches to isolation of patients as compared to no vaccine and controversy of isolation of HCV and HIV patients. In our center, the prevalence of HBV infection was 0% until 2005. The rate increased to 1.9% in 2006, 3.3% in 2007, 3% in 2008, 4.6% in 2009 and 13.7% in June 2010. The incidence rate was less than 2% per year. Therefore, in many units, patients on maintenance dialysis are still contracting new HBV infections and we need to be more careful about it. These patients require double dose of HBV vaccine (40 µg-20 µg in each deltoid muscles) scheduled at 0, 1, 2, and 6 month. Many physicians still continue to give three doses and all the 40 µg injected in a single deltoid despite clear mentioning of the dosage and schedule by the treating nephrologist. This requires awareness amongst non-nephrologists as large number of pre-dialysis patients with CKD get follow-up by physicians. Furthermore, not only physicians but also many nephrologists do not bother for assessing anti-HBs titer after vaccination to be sure of protection against HBV. Recently, I have seen new HBV infection developing in patients following renal transplantation suggesting that although they received transplants, they were still unprotected against HBV. Even when we get adequate vaccination, patients with CKD, with or without dialysis, experience lower seroconversion rates (32%-80%), lower peak antibody titers, and shorter durations of seroprotection (protective antibody titers maintained in 50% of patients with CKD compared with 85% of healthy individuals after one year). Therefore, these patients must be assessed by antibody titers regularly, probably once a year.Strategies to improve the response rate among such patients include vaccination as early as possible in the course of CKD. Additional strategies to improve vaccination response though important, still remain not well adapted. These include use of adjuvants to vaccine formulations to enhance the immunogenicity of the antigens. Aluminum is one such predominant adjuvant used in many currently available vaccines, including the standard HBV vaccine. Another adjuvant HB-AS04 has previously been demonstrated to have the ability to elicit higher and more persistent levels of anti-hepatitis B surface antibodies (anti-HBs) in CKD patients, when compared with double doses of standard HBV vaccine. While never tested previously in CKD patients, HB-AS02 has been shown to generate strong and persistent humoral and T-cell responses in healthy adults. The results of a recent meta-analysis also suggest significant benefits in the administration of levamizole as an adjuvant to HBV vaccine to increase seroprotection in patients with ESRD. Furthermore, in randomized controlled trial settings, both intradermal HBV vaccination and the addition of granulocyte-macrophage colony-stimulating factor (GM-CSF) have been shown to be better than standard HBV vaccination to generate a seroprotective antibody response. Our own study testing GM-CSF shows earlier protection with higher antibody titer in pre-dialysis patients with CKD (unpublished data). Moreover, these agents have been tested in cohorts comprising purely dialysis patients non-responsive to primary HBV vaccination. Each of these improvements has specific relevance to patients with CKD. Rapid seroconversion is clearly beneficial within the dialysis setting, as the sooner a protective antibody titer is obtained, the lower the cumulative risk of infection. The duration of protection afforded by HBV vaccination is directly proportional to the peak antibody level obtained after vaccination.The impact of renal transplant and treatment with immunosuppressive drugs on patients with HBV is not very clear. Immunosuppressive therapy is known to modify the natural history of the hepatic disease due to the increase in viral replication [6], though the real impact on patients' outcome in HBV-infected subjects does usually not become apparent in the initial few years of renal transplantation after which time liver failure is responsible for death in good number of cases [5]. Interferon used for the treatment of HBV infection, has been shown to be effective in large number of cases but in renal transplant patients it is contraindicated, as its immunomodulatory effects can induce rejection episodes. Of the other drugs being used for treatment of HBV, the maximum experience has been with Lamivudine-a synthetic nucleoside analogue with a potent action on HBV replication-with decreasing the progression of liver disease. Unlike interferon, Lamivudine does not have an immunomodulatory effect and therefore, could be safely used in renal transplant patients. We have recently published our experience of Lamivudine in these patients [7]. Though Entecavir and Tenofovir are promising new drugs for HBV infection, which are said to be better than Lamivudine for naive patients, their use in renal transplant patients is still to be studied. Till then, Lamivudine will continue to be a commonly used drug in these patients. Our experience showed that although HBV-PCR clearance is possible in nearly 50% of patients, HBe Ag and HBs Ag seroconversion is seen in very few patients. Enzyme normalization was seen in 77% of patients, though enzyme normalization is difficult to interpret in them, as many of these patients being immunosupprressed, may not show enzyme elevation in spite of having disease activity on histology. Practically, in these patients, we probably have to depend on virological response for assessing therapy rather than response to enzyme normalization. In terms of number of patients, our study is by far the largest number of patient series in renal transplant for assessing role of Lamivudine in these patients. Duration of treatment with Lamivudine is controversial. Since last year or so, we have started using Tenofovir in HBV patients on dialysis followed by renal transplant. Our initial impression is that it is better in terms of clearing the viral load. However, till now very few patients have been treated with Tenofovir and follow-up has also been short so making a definite conclusion is premature. Effect of treatment of HBV on patients in renal transplant setting in terms of liver outcome as well as outcome of transplant is not very well studies and reported. Our own study showed that the incidence of acute rejection in these patients is not different from untreated patients. However, long-term impact on graft and patients survival has not been commented upon in most of the studies. Assessing the outcome of liver, truly requires repeated liver biopsy to document regression, if any, of histological staging and grading. However, these types of studies are also lacking and with significantly decreasing prevalence of HBV in renal transplant patients, and short of multicenter studies, this information is unlikely to be available in near future.Treatment of HBV infection is changing with time. Unlike therapy with interferon which has a time bound schedule, duration of therapy with synthetic nucleoside analogues is not defined in either non-renal transplant or renal transplant patients. With the availability of newer drugs like Entecavir and Tenofovir, which have become first line therapy in naive HBV infected patients, we are likely to use these drugs in renal transplant patients also. However, in transplant patients it is time, which will uncover the efficacy and tolerability of these drugs, as in most of the clinical trials on these drugs, transplant patients have been excluded so that currently we can hardly find any studies with these drugs on renal transplant patients. Still, the best approach of management of these patients is vaccination against HBV with adequate dosage and assessment of the effectiveness of the vaccine along with isolation of infected patients during hemodialysis.
